# Uptake and Effects of the e-Vita Personal Health Record with Self-Management Support and Coaching, for Type 2 Diabetes Patients Treated in Primary Care

**DOI:** 10.1155/2016/5027356

**Published:** 2016-02-03

**Authors:** M. van Vugt, M. de Wit, F. Sieverink, Y. Roelofsen, S. H. Hendriks, H. J. G. Bilo, F. J. Snoek

**Affiliations:** ^1^Department of Medical Psychology, VU University Medical Center, 1081 HZ Amsterdam, Netherlands; ^2^EMGO^+^ Institute for Health and Care Research, VU University Medical Center, 1081 HZ Amsterdam, Netherlands; ^3^Centre for eHealth and Wellbeing Research, University of Twente, 7522 NB Enschede, Netherlands; ^4^Diabetes Centre, Isala, 8025 AB Zwolle, Netherlands; ^5^Department of Internal Medicine, University of Groningen and University Medical Center Groningen, 9713 GZ Groningen, Netherlands; ^6^Department of Medical Psychology, Academic Medical Center, 1105 AZ Amsterdam, Netherlands

## Abstract

We studied the use, uptake, and effects of e-Vita, a personal health record, with self-management support and personalized asynchronized coaching, for type 2 diabetes patients treated in primary care. Patients were invited by their practice nurse to join the study aimed at testing use and effects of a personal health record. Patients were followed up for 6 months. Uptake and usage were monitored using log data. Outcomes were self-reported diabetes self-care, diabetes-related distress, and emotional wellbeing. Patients' health status was collected from their medical chart. 132 patients agreed to participate in the study of which less than half (46.1%) did not return to the personal health record after 1st login. Only 5 patients used the self-management support program within the personal health record, 3 of whom asked a coach for feedback. Low use of the personal health record was registered. No statistical significant differences on any of the outcome measures were found between baseline and 6 month follow-up. This study showed minimal impact of implementing a personal health record including self-management support in primary diabetes care. Successful adoption of web-based platforms, as ongoing patient centered care, is hard to achieve without additional strategies aimed at enhancing patient motivation and engaging professionals.

## 1. Introduction

Type 2 diabetes mellitus (T2DM) is a chronic metabolic disorder characterized by hyperglycemia and an increased risk of developing micro- and macrovascular complications [1, 2]. The estimated world prevalence of 387 million T2DM patients is rapidly increasing [3]. To deal with the increasing number of people with T2DM, and burden on diabetes health care, alternative treatment options are being considered. Successful treatment of diabetes builds on empowering patients in their daily self-management of the disease, with a focus on healthy eating, being active, and taking medication as recommended [4–6]. A patient centered approach is called for to improve both medical and psychological outcomes [7–10]. Patient centered care is characterized by shared decision making between patient and professional, guided by the preferences, needs, and values of the patient [11]. One way of supporting patient centeredness is by using a personal health record (PHR) [12, 13]. In general, PHRs are web-portal environments with which patients can get an overview of their health outcomes, communicate with their care provider, and read information regarding their disease. PHRs support a patient centered approach by allowing patients to get more involved in their own disease management and decision making process. It has been shown that a PHR could be beneficial for people with T2DM [14]. Therefore, PHRs aimed at empowering patients with their self-care could have the potential of decreasing the workload of diabetes care providers and improve (cost-)effectiveness of diabetes treatment [15–18].

For these reasons, the foundation Care Within Reach (in Dutch: Stichting Zorg Binnen Bereik, founded by Philips and Achmea, a Dutch health insurance company) created the “e-Vita” PHR which advocates a patient centered approach for supporting people with T2DM who are treated in primary care in the Netherlands. Like comparable PHRs, e-Vita provides access to diabetes education and personal clinical outcome measures which are retrieved from the digital medical records of primary care practices. Additionally, e-Vita offers the opportunity of reading messages that were sent by the care provider and an additional self-management support program (SSP) [19]. An SSP is uncommon for PHRs and was added to further support patients in their diabetes self-management and to possibly uphold usage rates, which are known to be an issue for PHR [20, 21]. The SSP is based on the principles of personal goal setting and goal evaluation for behavioral change, guided by the Health Action Process Approach (HAPA) model from Schwarzer [22]. The SSP within e-Vita allows patients to choose from 4 predefined behavioral goals (diet, exercise, medication adherence, and stopping smoking) as advised by the Association of American Diabetes Educators (AADE) [5]. To support patients in achieving these goals, they can formulate self-chosen action plans, after which they are encouraged to carry them out. Eventually, patients are prompted to evaluate their behavioral goals and action plans with help from the SSP, based on graded tasks and barrier identification [23]. After goal evaluation, patients are encouraged to restart the behavioral goal setting and action planning procedure [21, 24]. Within the SSP, a coaching functionality was added, consisting of asynchronized messaging between coach and user, to enhance the effectiveness of the SSP and stimulate further continued usage of the e-Vita PHR [20].

In the current study, we looked at the uptake and effects of the e-Vita personal health record with self-management support program and additional asynchronized coaching, in a sample of type 2 diabetes patients treated in primary care.

## 2. Research Design and Methods

### 2.1. Design Overview

The scientific data comes from an overall 2-year e-Vita PHR project, which were made available for multiple research institutions to conduct longitudinal cohort studies and (cost-)effectiveness studies [19, 25]. Data for this study were obtained from a randomized controlled trial (RCT) with the e-Vita PHR and the SSP, which was part of the bigger overall 2-year e-Vita PHR project [21]. The study was approved by the medical ethical committee of the VU University Medical Center.

### 2.2. Setting and Participants

Participants for the e-Vita project were approached within 52 primary health care practices with the possibility of reaching approximately 8300 T2DM patients for a study period of 2 years. For the current study, participants were enrolled for an inclusion period of 6 months between July 1, 2013, and December 31, 2013. Patients who visited their primary care physician for routine checkup were made aware of the study and the availability of the PHR by their practice nurse. Patients received information about the study, and if they agreed to participate, they had to sign an informed consent and fill out questionnaires at different time-points during the study period. When patients expressed interest in using the PHR, the practice nurse registered the patient into the PHR (online registration) and provided a brochure with information regarding the login procedure. After registration, the patient received automated login codes via e-mail. Patients could use the PHR, without having to participate in the study.

In general, the health care practitioners who agreed to facilitate the study and PHR received financial compensation if they were able to include patients in the overall e-Vita project. However, there were no direct incentives for patients nor professionals to participate. By doing so, we tried to resemble standard care as much as possible. Inclusion criteria were a diagnosis of T2DM and age of ≥18 years. Exclusion criteria were mental retardation or psychiatric treatment for schizophrenia, organic mental disorder, or bipolar disorder currently or in the past, insufficient knowledge of the Dutch language, life expectancy of <1 year due to malignancies or other terminal illnesses, and/or cognitive impairment.

### 2.3. Coaching

Between July 1, 2013, and December 31, 2013, patients who logged into the PHR for the first time were informed about a study and were asked for consent to participate in the study, by selecting an option “yes I agree to participate.” Patients were able to use the PHR without being randomized, and then they would not be included in the current study. After consent, participants were randomized into 2 groups. Participants remained blinded for group allocation. Some participants were able to ask for feedback from a coach after they had set a goal and planned an action within the SSP (coaching group; CG) and others could not (noncoaching group: NCG). The feedback of the coach would mainly contain positive appraisal and constructive advice for improving the planned action of the patient by commenting on specificity, measurability, attainability, realism, and the time frame. Additionally, participants received personal messages from their coach, which consisted of one welcome message (0 weeks) and 2 encouraging reminders at 4 weeks and 8 weeks after enrollment to keep using the PHR and the SSP. All messages contained additional instructions on how to use the SSP within the PHR.

### 2.4. Measurements

The* use* of the PHR and the SSP was tracked objectively by collecting anonymized log data, which contained information about time, date, and type of actions performed within the PHR.

For baseline (T0) and follow-up measurements after 6 months (T1), the following information was obtained.

*Diabetes self-care* (general diet, specific diet, fruit intake, carbohydrate intake, fat intake, 30 minutes of exercise behavior, specific workouts, blood glucose control, medication adherence, foot care, and shoe check-up) was measured by the Summary of Diabetes Self-Care Activities (SDSCA), measured on an 8-point scale (*α* = .47) generating mean scores ranging from 0 to 7 days a week [26, 27].

*Diabetes-related distress* was assessed by the Problem Areas In Diabetes care survey 5-item version (PAID-5), measured on a 5-point Likert scale (*α* = .86) with total sum score ranging from 0 to 20, where elevated distress is defined by scores >8 [28].

*Emotional wellbeing* was measured with the World Health Organization Wellbeing Index 5 items' questionnaire, measured on a 5-point Likert scale (*α* = .86). The total sum scores are transferred from 0 to 100, where higher scores indicate better mood [29, 30].

*Health status* (glycemic control (HbA1c), Body Mass Index (BMI), systolic blood pressure, diastolic blood pressure, cholesterol, and smoking status) was extracted from patients' health care records, covering the same time period of when patients participated in the study. Additionally social demographic information was obtained (gender, age, education, occupation, and prescribed medication).

### 2.5. Statistical Analysis

Percentages were calculated to examine login, use of the PHR, SSP, and coaching functionality. Analyses were conducted using SPSS software. We applied a two-sided 5% level of significance for all statistical analyses. Longitudinal linear regression, using Generalized Estimation Equations (GEE), was applied to investigate the differences on outcome variables over time and between the two groups. Analyses were based on intention-to-treat. All analyses were corrected for age, gender, T2DM duration, complications, ethnicity, and outcome baseline values.

## 3. Results

### 3.1. Inclusion

In the overall e-Vita project, 1378 patients participated, 947 of which expressed interest in using the PHR, and 405 patients were eventually registered by the practice nurse to use the PHR [25]. For the current study, from July 2013 until December 2013, 165 people were registered by their practice nurse to use the PHR, of which 132 (80%) agreed to participate in the current study. Of the 132 people who agreed, 66 (50%) were able to use the coaching functionality within the SSP. More than half of the participants were male (59.1%). Mean age was 67.9 (SD = 10.4). The baseline sociodemographic, clinical, and medical characteristics of the study sample are summarized in Table 1.

### 3.2. Use

During the period from July 2013 to July 2014, 128 (96.9%) participants logged into the PHR after randomization and inclusion. Of these 128 people, 59 (46.1%) participants (28 CG and 31 NCG) never returned to the PHR during the study period. An overview of frequencies of the number of logins is presented in Table 2. Six participants (5 CG and 1 NCG) used the SSP within the PHR. The demographic information of these 6 people is shown in Table 3. Three participants used the coaching functionality within the SSP and asked for feedback on their set goals. Their goals can be grouped into* healthy eating* (*n* = 3),* being active* (*n* = 3), and* quitting smoking* (*n* = 1).  Table 4 shows the actions per session of the three participants who asked for feedback. In general, 1 participant used the SSP in combination with the* overview of personal clinical outcome measures*, while 1 participant used the SSP in combination with the* diabetes education*. One participant only used the SSP without using other components.

Participants in the coaching group received 3 additional personal messages from their coach in the form of a welcome message and 2 reminders. 16.7% logged in within one week after receiving the welcome message, compared to 4.5% of the NCG. 9.1% logged in within one week after the first reminder, compared to 7.6% of the NCG. 15.2% logged in within one week after the second reminder, compared to 7.6% of the NCG, who did not receive a welcome message or reminders. The number of logins after the reminder messages is presented in Table 5. Throughout the study period, 2 e-mail messages with news updates were sent from the e-Vita PHR to all 132 participants of this study. 82.9% of the participants logged in within one week after receiving the first general message (85% CG and 80.9% NCG). 31.8% of the participants logged in within one week after receiving the second general message (25% CG and 41% NCG).

### 3.3. Outcome Measures

A total of 68 participants (51.5%) filled in the follow-up questionnaire (T1). For these participants (CG: 29, NCG: 39), statistical analyses showed that there were no significant differences in time on any of the outcome measures between baseline and T1 follow-up for the two groups.

## 4. Discussion

The aim of this study was to assess the uptake and effects of a personal health record with a self-management support program and additional asynchronized coaching, for type 2 diabetes patients treated in primary care. Our most important findings are discussed below.

### 4.1. Inclusion of Patients

The inclusion rate of the current study was dependent on the overall 2-year e-Vita PHR project. As mentioned by Roelofsen et al., the inclusion rate of participants for the overall 2-year e-Vita PHR project turned out to be lower than anticipated, which may have had influence on the inclusion of the current study. In the overall e-Vita project, 70.6% of the approached patients were interested in using the PHR. However, only 42% of these patients were enrolled by their care provider [25]. The care providers involved in the e-Vita project indicated that lack of integration of the PHR with work routines, lack of knowledge about the PHR, lack of time, and PHR related usability problems were the main reasons for not using the PHR in daily routine care and not referring or enrolling patients [31]. Eventually, for the 2-year e-Vita project, only 27% of people who were registered to use e-Vita logged in at least once. It was later uncovered that difficult login procedures with e-Vita may have discouraged patients to log in [31]. Therefore, it was possible that patients with low technological skills may not have been included in the current study.

### 4.2. Usage of the Personal Health Record

When looking at the usage of the PHR for people in the current study, the initial high login rate may indicate that patients were interested in using the PHR, which seems in line with recent research, which shows that the older population is increasingly using the Internet to maintain their independence [32]. However, the rapidly declining use could indicate that the aim of the e-Vita PHR, which was to support patient centeredness and promote healthy behavioral change, may not have matched the expectations or needs of the patients [24]. It could be that patients are not yet ready to embrace a patient centered approach and therefore do not feel compelled to use the PHR. The low usage may also indicate that the content of the PHR was not sufficient to support patient centeredness or not appealing enough to stimulate continued usage. Forgetting about the PHR can contribute to underuse as well [33]. Sending multiple personal and general messages to stimulate use of the PHR and the SSP did seem to influence some people to log in again but did not result in a substantial increase of usage of the SSP.

Research has shown that a perceived positive health status by patients may contribute to low use of a PHR [33]. The outcome measures in this study indicated that, besides BMI, patients were well controlled and had little room for improvements (e.g., glycemic control < 50 mmol/mol; cholesterol < 4.5 mmol/L; diastolic blood pressure < 80 mm Hg). This positive health status may have lowered the patients' perceived need for continuously using a PHR.

It is known that professional caregiver endorsement plays a vital role in encouraging patients to use the PHR [34]. Interviews with care providers revealed that they did not embrace using the PHR in their work routines [31]. Due to the relatively high quality of primary care and well-controlled T2DM patients in the Netherlands, it could be that care providers simply do not feel the need to integrate a PHR in daily care routines or recommend it to their patients.

### 4.3. Usage of the Self-Management Support Program

The SSP was developed to help sustain usage and to support patients with changing their health behaviors by endorsing goal setting and action planning. The well-controlled health status of the patients, and possible absence of perceived disease burden, may have contributed to low intentions for behavioral change and subsequent low usage of the SSP. When patients do not have intentions for behavioral change, then goal setting and action planning might not be considered as relevant or useful [35]. Therefore, at this stage, the SSP might be a mismatch with the needs and expectations of the patients who agreed to use the PHR. Interestingly, the clinical profiles of the 3 patients who did actively use the SSP did not indicate that they would highly benefit from using the SSP.

However, these patients had been recently diagnosed with T2DM. It could be that these patients were still adapting to their diagnosis and looking for information on effective coping strategies. For the SSP to be used more, it will need to match patients' needs and intentions for behavior change and should be further endorsed by the care provider. The lack of engagement and high attrition have been observed repeatedly in e-health. Most promising remedy appears to be “blending” of e-health with face-to-face consultations, thus affecting involvement of professionals. Having only the PHR target patients' risk perception, self-efficacy, and outcome expectancies, which are determinants of intention formation (motivation) for behavior change, does not seem to guarantee engagement in using the PHR for healthy behavior change. Both care provider and PHR should facilitate intention formation, by raising risk awareness, and increase outcome expectancy and self-efficacy [22].

Finally, the underuse of the SSP could also indicate that the “look and feel” was not attractive enough to stimulate use. The SSP may have contained too few introductory texts and may have not always been as intuitive in use.

### 4.4. Development and Implementation

The initial development and implementation protocol of e-Vita followed a linear process, in which patient focus groups were held but where pilot testing and development feedback loops were absent. Additionally, the study protocol required a controlled condition, which hampered the flexibility of the development process. The linear development process and initial lack of pilot testing before implementation could have caused a mismatch with patients' needs, which may have contributed to the underuse of the SSP in this study [36]. Currently, after the completion of this study, the development and implementation process adapted towards an iterative process, following a sequential process of development, feasibility and pilot testing, evaluation, and implementation, which is in line with the Medical Research Council (MRC) framework for complex interventions [37]. For future studies on PHRs, the Medical Research Council (MRC) framework for complex interventions could offer a solution for guiding development, implementation, and complex study processes [37].

### 4.5. Effectiveness of the Personal Health Record

Only 68 (51.6%) of the 132 users filled in the T1 follow-up measurements after 6 months, which hampered testing of program and coaching effectiveness. There were no differences in outcome measures over time, nor were there differences between the coaching and noncoaching groups. We analyzed the use of the PHR by the three users who asked their coach for feedback; however, the sample was too small to make statements on the effects of a PHR with additional asynchronized coaching.

## 5. Conclusion

To successfully implement a PHR in a standard care setting, both care provider and patient will need to see the added value and engage actively in the process. In this study, the introduction of the PHR clearly had little impact and was not yet fully integrated into the clinical routine. Future studies should explore ways to effectively prepare both patients and professionals, building on principles of patient centeredness and self-management. Furthermore, for facilitating the use of self-management support programs within a PHR, patients first need to develop intentions for behavioral change, which can only be achieved if patients have sufficient risk awareness, experience a need for behavioral change, and feel self-confident in making these changes. To ensure uptake and effectiveness of a PHR in health care, an iterative process of continued development, feasibility and pilot testing, and evaluation is important.

## Figures and Tables

**Table 1:** Baseline characteristics.

	Total (*n* = 132)	CG (*n* = 66)	NCG (*n* = 66)	*P* value
*Sociodemographics*				
Gender				.239
Female	54 (40.9%)	37 (56.1%)	25 (37.9%)	
Male	78 (59.1%)	29 (43.9%)	41 (62.1%)	
Age	67.9 (10.4)	67.4 (10.5)	68.3 (10.4)	.602
<50	6	2	4	
50–64	47	30	17	
65–74	50	18	32	
>75	29	16	13	
Ethnicity^1^				1.000
Caucasian	91	45	46	
Non-Caucasian	1	1		
Education^2^				.866
No or school level qualifications	20 (15.2%)	10 (15.2%)	10 (15.2%)	
Professional or vocational	46 (34.8%)	21 (31.8%)	25 (37.9%)	
Bachelor's degree or higher	43 (32.6%)	22 (33.3%)	21 (31.8%)	
Employed	43 (32.6%)	18 (27.3%)	25 (36.9%)	.529
Medical outcomes				
Diabetes duration	5.82 (±4.62)	5.77 (±4.35)	5.86 (±4.91)	.917
BMI	30.19 (±5.16)	30.72 (±5.06)	29.67 (±5.25)	.284
HbA1c (mmol/mol)	48.50 (±7.49)	48.49 (±7.31)	48.52 (±7.33)	.985
Treated with tablets^3^	87 (65.9%)	44 (66.7%)	43 (65.2%)	.652
Treated with insulin^3^	15 (11.4%)	10 (15.2%)	5 (7.6%)	.172
Treated with tablets and insulin^3^	14 (10.6%)	9 (13.6%)	5 (7.6%)	.263
Systolic blood pressure (mm Hg)	135.57 (±15.58)	136.65 (±16.62)	134.53 (±14.58)	.473
Diastolic blood pressure (mm Hg)	78.47 (±9.58)	78.07 (±9.48)	78.86 (±9.74)	.670
Cholesterol (mmol/L)	4.34 (±.84)	4.19 (±.83)	4.50 (±.82)	.045
Smoking^4^	18 (13.6%)	9 (13.6%)	9 (13.6%)	.954
Outcome measures				
Emotional wellbeing	70.83 (±14.84)	71.21 (±13.02)	70.47 (±16.46)	.798
Diabetes distress	2.15 (±2.41)	2.37 (±2.51)	1.96 (±2.32)	.385
General diet	5.59 (±1.83)	5.88 (±1.57)	5.30 (±2.02)	.102
Specific diet	4.44 (±.84)	4.46 (±.83)	4.42 (±.86)	.814
Exercise	3.91 (±1.76)	3.90 (±1.95)	3.92 (±1.58)	.964
Foot care	1.80 (±2.13)	1.93 (±2.11)	1.68 (±2.15)	.542

*Note*. CG: coaching group; NCG: noncoaching group; BMI: Body Mass Index; HbA1c: blood glucose control; ^1^ *n* = 40 missing data; ^2^ *n* = 23 missing data; ^3^ *n* = 20 missing data; ^4^ *n* = 17 missing data.

**Table 2:** Login frequency of participants in the RCT study.

Number of logins	Total (*n* = 132)		CG (*n* = 66)		NCG (*n* = 66)	
	Users who logged in	Average duration (m:s.ms)	Users who logged in	Average duration (m:s.ms)	Users who logged in	Average duration (m:s.ms)
1	128 (97.0%)	08:47.87	65 (98.5%)	10:18.06	63 (95.5%)	07:12.78
2	69 (52.3%)	07:50.90	37 (56.1%)	07:50.59	32 (48.5%)	07:51.25
3	44 (33.3%)	11:06.41	22 (33.3%)	15:03.73	22 (33.3%)	07:09.09
4	31 (23.5%)	10:28.03	18 (27.3%)	14:50.28	13 (19.7%)	04:24.92
5	24 (18.2%)	11:38.08	14 (21.2%)	10:00.00	10 (15.2%)	13:55.40
6	18 (13.6%)	07:11.56	12 (18.2%)	07:48.67	6 (9.1%)	05:57.33
7	17 (12.9%)	09:17.76	11 (16.7%)	08:06.18	6 (9.1%)	11:29.00
8	13 (9.8%)	06:55.85	8 (12.1%)	09:10.25	5 (7.6%)	03:20.80
9	10 (7.6%)	08:39.40	6 (9.1%)	07:15.50	4 (6.1%)	10:45.25
10	10 (7.6%)	12:09.60	6 (9.1%)	09:52.00	4 (6.1%)	15:36.00
11	8 (6.1%)	03:52.75	4 (6.1%)	02:59.00	4 (6.1%)	04:46.50
12	8 (6.1%)	06:09.37	4 (6.1%)	07:45.75	4 (6.1%)	04:33.00
13	6 (4.5%)	14:17.33	3 (4.5%)	23:58.33	3 (4.5%)	04:36.33
14	5 (3.8%)	03:37.00	3 (4.5%)	01:40.67	2 (3.0%)	06:31.50
15	3 (2.3%)	03:23.67	1 (1.5%)	01:00.00	2 (3.0%)	04:35.50
16	2 (1.5%)	26:19.50	1 (1.5%)	08:24.99	1 (1.5%)	44:14.00
17	1 (0.8%)	01:00.00	0		1 (1.5%)	01:00.00
18	1 (0.8%)	01:00.00	0		1 (1.5%)	01:00.00
19	1 (0.8%)	02:21.00	0		1 (1.5%)	02:21.00
20	1 (0.8%)	02:12.00	0		1 (1.5%)	02:12.00
21	1 (0.8%)	01:00.00	0		1 (1.5%)	01:00.00
22	1 (0.8%)	01:00.00	0		1 (1.5%)	01:00.00
23	1 (0.8%)	06:49.00	0		1 (1.5%)	06:49.00
24	1 (0.8%)	01:00.00	0		1 (1.5%)	01:00.00
25	1 (0.8%)	04:16.00	0		1 (1.5%)	04:16.00

*Note*. CG: coaching group; NCG: noncoaching group; m: minutes; s: seconds; ms: milliseconds.

**Table 3:** Baseline characteristics of participants who used the self-management support module.

	User 1	User 2	User 3	User 4	User 5	User 6
Group	CG	CG	CG	CG	CG	NCG
Planned action and asked for feedback	Yes (2x)	Yes (3x)	Yes (2x)	No	No	No
Range of platform use from 1st login (weeks)	7	26	11	11	0	0
Sociodemographics						
Gender	Female	Female	Female	Female	Female	Male
Age	40	45	58	71	57	57
Ethnicity	White	White	—	White	—	White
Education	—	BScMSc	BScMSc	SLQ	Prof/voc	BScMSc
Employment	—	Full time	Part time	Retired	Part time	Unemployed
Medical characteristics						
BMI	30.11	26.33	23.34	43.12	—	34.72
HbA1c mmol/mol	41	43	47	50	—	43
HbA1c %	5.9	6.1	6.5	6.7	—	6.1
Diabetes duration in years	2	1	1	6	—	16
Treatment	Tablets	Tablets	Tablets	Tablets	—	Insulin/tablets
Psychological characteristics T0						
WHO5	—	68	80	64	72	92
PAID5	—	9	2	0	5	2
Behavioral characteristics T0						
General diet	—	6	7	7	3.5	5
Specific diet	—	4.67	4.67	5.33	5	6.33
Exercise	—	5.0	1.5	2.5	4	5.5
Medication adherence	—	7	7	7	—	7
Foot care	—	2	0	7	3.5	.5
Self-monitoring blood glucose	—	1.5	—	—	—	.5

*Note*. CG: coaching group; NCG: noncoaching group; BMI: Body Mass Index; HbA1c: blood glucose control; WHO5: World Health Organization 5 questionnaire; PAID5: problem areas in diabetes questionnaire; —: missing data.

**Table 4:** Actions per session of the three participants who asked for feedback.

User	Session	Used component within the PHR
User 1	8-Aug-13	Education
	4-Sep-13	Yearly checkups + education (9 topics, 13 views)
	6-Sep-13	Education (35 topics) + adding goal, action (*healthy eating, being active*) + education
	6-Sep-13	Yearly checkups + goals + information + education (2 topics)
	6-Sep-13	Reading feedback coach
	6-Sep-13	Yearly checkups + evaluating action + adding new goal, action
	4-Oct-13	Reading feedback coach
	15-Oct-13	Monitoring weight + BMI + yearly checkups
	10-Nov-13	Monitoring weight + BMI + yearly checkups + adding goal evaluation (incl. coaching feedback)
	14-Nov-13	Home
	18-Dec-13	Monitoring weight + BMI + waist circumference + yearly checkup
	16-Jan-14	Monitoring weight + blood pressure + yearly checkup + monitoring BMI
	9-Feb-14	Monitoring weight + yearly checkups
	22-Feb-14	Yearly checkups HbA1c
	28-Jun-14	Monitoring weight + yearly checkups + extra information + education (1 topic)
	5-Aug-14	Monitoring weight (BMI)
	6-Aug-14	Yearly checkups
	22-Aug-14	Coaching

User 2	31-Aug-13	Home + yearly checkups + coaching
	31-Aug-13	Education + yearly checkups
	8-Sep-13	Adding goals, action (*healthy eating*, *being active*, and *quitting smoking*) + education (5 topics)
	5-Oct-13	Adding evaluation; monitoring blood pressure
	7-Oct-13	Reading feedback coach
	23-Oct-13	Overview goals; monitoring blood pressure + yearly checkups

User 3	30-Dec-13	Education
	31-Dec-13	Yearly checkups + monitoring + extra information
	2-Jan-14	Home
	7-Feb-14	Goals + education (3 topics) + messages + yearly checkups + education (6 topics) + goals + extra information + education + extra information + goals + extra information + education + adding goals, actions (*healthy eating*, *being active)*
	7-Feb-14	Reading feedback coach + education
	11-Feb-14	Coaching + education (4 topics)
	27-Feb-14	Evaluating action (not added) + education (3 topics)
	8-Mar-14	Education (5 topics) + coaching + education (1 topic) + goals + coaching Button + extra information
	19-Mar-14	Home
	14-May-14	Education
	24-Aug-14	Home
	20-Sep-14	Explanation AlbCreatRatio; Cockroft

*Note*. A session is defined as a unique and new login moment.

**Table 5:** Number of people logged in within a week after a reminder or message.

Number of people logged in	Total (*n* = 132)	CG (*n* = 66)	NCG (*n* = 66)
PHR e-mail 1 (24-7-2013)	**34/132 (25.8%)**	**17/66 (25.8%)**	**17/66 (25.8%)**
	34/41 (82.9%)	17/20 (85.0%)	17/21 (81.0%)
PHR e-mail 2 (21-10-2013)	**29/132 (22.0%)**	**13/66 (19.7%)**	**16/66 (24.2%)**
	29/91 (31.9%)	13/52 (25.0%)	16/39 (41.0%)
Welcome message (IG only, sent immediately after 1st login)	14 (10.6%)	11 (16.7%)	3 (4.5%)
Reminder 1 (IG only, sent 4 weeks after 1st login)	11 (8.3%)	6 (9.1%)	5 (7.6%)
Reminder 2 (IG only, sent 12 weeks after 1st login)	15 (11.4%)	10 (15.2%)	5 (7.6%)
Platform use in weeks	9.75 (8.48)	9.97 (8.53)	9.50 (8.55)

*Note*. CG: coaching group; NCG: noncoaching group. At the moment of sending the e-mail messages, not all 132 participants were registered yet; e-mail 1 was sent to a total of 41 participants; e-mail 2 was sent to a total of 91 participants.
